# The impact of skin-to-skin contact upon stress in preterm infants in a neonatal intensive care unit

**DOI:** 10.3389/fped.2024.1467500

**Published:** 2024-11-08

**Authors:** Halyna Pavlyshyn, Iryna Sarapuk, Uliana Saturska

**Affiliations:** Department of Pediatrics No2, I. Horbachevsky Ternopil National Medical University, Ternopil, Ukraine

**Keywords:** preterm infants, NICU-related stress, cortisol, melatonin, skin-to-skin contact

## Abstract

**Introduction:**

Neonatal stress significantly affects the early adaptation, maturation and long-term development of preterm infants.

**The objective of the study:**

To investigate the effect of skin-to-skin contact (SSC) on stress level in preterm infants.

**Materials and methods:**

The research was a prospective study. Stress indicators (cortisol, melatonin) were measured before the SSC began (pre-intervention level) and after this intervention (post-intervention).

**Results:**

The study included 150 preterm infants in the NICU with gestational age (GA) ≤36 weeks. Pre-intervention salivary cortisol level was higher in extremely and very preterm neonates compared to moderate and late preterm newborns (*p* = 0.028), in children with low Apgar scores (*p* = 0.041), in those who were on mechanical ventilation (*p* = 0.005), and suffered neonatal sepsis (*p* = 0.005). Pre-intervention melatonin level was lower in children with low Apgar scores (*p* = 0.032). Salivary cortisol levels were significantly decreased after SSC in preterm infants [pre-intervention: 0.294 (0.111; 0.854) μg/dL vs. post-intervention: 0.127 (0.070; 0.229) μg/dL, *p* < 0.001], and urinary melatonin levels were significantly increased after SSC [pre-intervention: 4.01 (2.48; 6.34) ng/mL vs. post-intervention: 5.48 (3.39; 9.17) ng/mL, *p* < 0.001]. A greater reduction in cortisol levels after skin-to-skin contact was revealed in infants with a lower gestational age (*p* = 0.022), in boys compared to girls (*p* = 0.012), in infants with respiratory distress syndrome (*p* = 0.048), in those who had mechanical ventilation compared to non-ventilated neonates (*p* = 0.008), and in infants with seizures (*p* = 0.036). The melatonin levels increased more intensively in infants with low Apgar scores (*p* = 0.002), and in those with late-onset sepsis (*p* = 0.006).

**Conclusion:**

The reduction in cortisol levels and the increase in melatonin levels provided strong evidence that SSC ameliorated the NICU-related stress in preterm infants. We found higher indicators of stress and more dramatic responses to SSC in reducing indicators of stress in infants with lower GA than in infants with higher GA, indicating that SSC may be even more important for lower GA infants. The infants who need SSC the most should not be denied the care they need to reduce the stress they experience from being born too soon and continuing their gestational development in the stressful environment of the NICU.

## Introduction

Preterm infants are especially vulnerable to stress due to the morphological and functional immaturity of their systems and organs (1). Stress significantly affects the early adaptation, maturation and long-term development of premature neonates. It can hinder brain development, leading to cognitive, behavioral and emotional problems, and impaired visual abilities (2, 3). Chronic stress slows growth and reduces the protective properties of the immune system that makes preterm children more vulnerable to infections and diseases (4). Additionally, the immature organism's response to stress can manifest in physiological instability, such as irregular breathing and heartbeats, and drops in oxygen saturation, which threatens their survival (5).

Immature preterm infants in the neonatal intensive care unit (NICU) are exposed to many factors that induce stress and pain. These include frequent painful medical interventions, such as blood sampling, respiratory support and provision of infusion therapy; high levels of noise and light; limited physical contact with parents; feeding difficulties; higher risk of infections and other medical complications (2, 6, 7).

Recognizing stress in preterm infants is critical for timely intervention. The most common markers include changes in physiological indicators (fluctuations in heart rate, respiratory rate, blood pressure, and blood oxygen saturation), behavioral signs (irritability, crying, and sleep disturbances), physical reactions (facial grimaces, extension of limbs, and arching of the back, etc.). Biochemical markers of stress and pain increased level of adrenaline, norepinephrine, endorphins, aldosterone, antidiuretic hormone, cortisol, and glucagon, as well as decreased insulin secretion (8–10). According to recent experimental findings, stress-related events are also associated with melatonin alterations (11).

Coping with stress is vital for the preterm infants for their short- and long-term outcomes. To prevent the harmful effects of stress in preterm infants, various methods of stress reduction are used in NICUs (12, 13). Modifying the environment by reducing the level of noise and light in the NICU, using covers for incubators and creating an environment similar to the mother's womb can diminish stress for these tiny neonates, promotes their better neurodevelopment, and stimulates the production of anti-stress hormones, particularly melatonin (14). Cluster care, a modern treatment method that involves performing all the necessary medical interventions in single session to ensure longer periods of undisturbed rest proven to be highly effective (15, 16). The use of analgesics, non-pharmacological methods of pain and stress management, and glucose solution are recommended for newborns during painful procedures (17, 18).

Encouraging parents to participate in infant care, such as feeding, bathing and soothing, also has a pronounced protective, stress-buffering effect, promoting better development (19, 20). Today, scientists from different countries have observed high efficiency of skin-to-skin contact between preterm neonates and parents. It is one of the most effective methods of reducing stress, stabilizing the heartbeat, improving sleep and strengthening the psycho-emotional bonding (21, 22).

### The objective of the study

To investigate the effect of skin-to-skin contact on stress level in preterm infants.

## Materials and methods

### Study design

The research was a prospective study. Stress indicators (cortisol and melatonin) were measured before the initiation of skin-to-skin contact between the baby and the mother (baseline level of hormones – pre-intervention) and after this intervention (post-intervention hormone levels) in the NICU. Skin-to-skin contact (SSC) lasted a minimum of 60 min and more. The intervention was provided on the 3rd-10th day of the infant's life.

Pre-intervention sample collection was before initiation of skin-to-skin contact, post-intervention samples collection – after skin-to-skin contact (daily SSC for a min of 60 min for 3–5 days in a row). Post-intervention samples were collected exactly after the SSC episode (saliva samples were collected immediately and urine samples – in two hours after SSC).

Research was conducted in a regional tertiary care unit housed within a perinatal center. All infants in this unit were placed in an open-bay NICU architecture. Parents had 24/7 access to the NICU, participate in infant care and have possibilities to perform skin-to-skin contact with their infants. There was noise and light control, incubators were protected with dark covers, medical staff spoke quietly, and mobile phones were not used in the NICU. Breastfeeding was encouraged.

Naked preterm newborns were placed on the mother's chest dressed only in a hat and nappy. Skin-to-skin contact was performed for all hemodynamically stable infants, including those on mechanical ventilation.

### Sample collection and stress markers assay

Level of neonatal stress before and after skin-to-skin contact in preterm infants was evaluated by measuring salivary cortisol and urinary melatonin levels. Urine and saliva samples were collected using cotton sponges, which were then, centrifuged (2 min at 2000× g). Saliva samples were collected without the use of any salivation-stimulating agents. After extraction, saliva samples were frozen and stored at −20°C, urine samples were centrifuged for 20 min at 1000× g at 2–8°C then frozen and stored at −80°C. Cortisol levels in the saliva samples were analyzed using an enzyme immunoassay for the quantitative determination of free cortisol in human saliva (IBL International GmbH, Hamburg, Germany). Melatonin levels in the urine samples were analyzed using an enzyme immunoassay for the quantitative determination of melatonin sulfate (Elabscience, Wuhan, China). Samples were analyzed in duplicate, and assays were performed using provided controls.

### Human research statement

The study was approved by the local ethical committee (I. Horbachevsky Ternopil National Medical University), and informed consent was obtained from all study mothers for themselves and for their infants. The research was conducted in accordance with the World Medical Association's Helsinki Declaration.

### Statistics

All computations were performed using StatSoft STATISTICA Version 13 (Tulsa, OK). Quantitative data were presented as the median and interquartile range (IQR; 25th to 75th percentiles). For qualitative parameters, absolute and relative frequencies were presented. Proportions were compared between the two groups using the two-tailed Fisher exact test. Spearman correlations were used to assess the associations among measures. Wilcoxon matched pairs test (for two dependent groups) was used to identify differences in pre-post levels of laboratory markers. Significance was assumed at *p* < 0.05 level. The required sample size was calculated using G*Power Software sample size calculator.

## Results

### Recruitment and randomization

There were 172 eligible infants, with 157 ultimately recruited. Three infants did not meet the inclusion criteria, five neonates were excluded due to insufficient investigated sample (saliva), and seven parents declined to participate. The inclusion criteria were prematurity [gestational age (GA) ≤36 weeks] and maternal willingness to perform skin-to-skin contact after signing informed consent. Exclusion criteria included congenital abnormalities, chromosomal diseases, and congenital specific infection (human immunodeficiency virus, toxoplasmosis, rubella, cytomegalovirus, and herpes).

Out of the 157 recruited infants, seven mothers did not perform skin-to-skin contact with their children due to maternal reasons. Thus, the data of 150 preterm infants were analysed.

### Clinical characteristics of the study group

A total of 87 (58.0%) extremely and very preterm infants (GA < 32 weeks) and 63 (42.0%) moderately and late preterm infants (GA > 32 weeks) were examined. There were 78 (52.0%) boys, 66 (48.0%) girls, with no significant difference depending on the GA of the children (*р* = 0.790). There were 50 twins (33.3%) and 100 babies born from a singleton pregnancy (66.7%). The mean GA of extremely and very preterm infants was 30 (±2.0) weeks, and the mean GA for moderate and late preterm infants was 33.8 (±0.9) weeks.

No significant differences were found in the perinatal risk factors between infants with GA of less than 32 weeks and those with a GA of more than 32 weeks, except for gestational hypertension and preeclampsia, which significantly prevailed in the history of moderately and late preterm infants (42.9% vs. 24.1%, *p* = 0.021). One hundred ten preterm neonates (73.3%) were born by caesarean section. There was no significant difference in the frequency of vaginal delivery and caesarean section in groups of children with different GA, *p* > 0.05. The specific details of pregnancy and delivery history are presented in Table 1.

**Table 1:** Characteristics of infants of the study group.

Indicator	Statistical indicator	Extremely and very preterm infants, *n* = 87	Moderate and late preterm infant, *n* = 63	*р*
History of pregnancy and delivery				
Preeclampsia, eclampsia, gestational hypertension	*n* (%)	21 (24.1%)	27 (42.9%)	*p* = 0.021
Thyroid gland disorders	*n* (%)	10 (11.5%)	6 (9.5%)	*p* = 0.458
Placental dysfunction	*n* (%)	56 (64.4%)	46 (73.0%)	*p* = 0.173
Anemia	*n* (%)	40 (46.0%)	25 (39.7%)	*p* = 0.274
Urinary tract infection	*n* (%)	10 (11.5%)	12 (19.0%)	*p* = 0.145
Multiple pregnancies	*n* (%)	27 (31.0%)	23 (36.5%)	*p* = 0.299
Clinical characteristics				
Apgar score at 1st min	Me [Lq; Uq])	6.0 [5.0; 7.0]	7.0 [7.0; 7.0]	*p* < 0.001*
Apgar score at 5th min	Me [Lq; Uq])	7.0 [7.0; 7.0]	7.0 [7.0; 7.0]	*p* = 0.002*
Birth weight, g	Mean ± SD	1,461.40 ± 371.15	1,950.57 ± 438.30	*р* < 0.001*
Birth length, cm	Mean ± SD	38.93 ± 3.30	43.44 ± 2.81	*р* < 0.001*
Head circumference, cm	Mean ± SD	28.49 ± 2.39	30.51 ± 1.79	*р* < 0.001*
RDS	*n* (%)	75 (86.2)	28 (44.4)	*р* < 0.001*
Surfactant replacement therapy	*n* (%)	36 (41.4)	1 (1.6)	*р* < 0.001*
Mild and moderate respiratory disorders	*n* (%)	54 (62.1)	55 (87.3)	*р* < 0.001*
Severe respiratory disorders	*n* (%)	33 (37.9)	8 (12.7)	
Early neonatal sepsis	*n* (%)	28 (32.2)	17 (27.0)	*p* = 0.308
Hypoxic-ischemic encephalopathy II-III	*n* (%)	17 (19.5)	8 (12.7)	*p* = 0.188
Intraventricular hemorrhages	*n* (%)	20 (23.0)	11 (17.5)	*p* = 0.269
Neonatal seizures	*n* (%)	18 (20.7)	6 (9.5)	*p* = 0.051
Late-onset neonatal sepsis	*n* (%)	17 (19.5)	4 (6.3)	*p* = 0.017*
Necrotizing enterocolitis	*n* (%)	20 (23.0)	9 (14.3)	*p* = 0.130
Mechanical ventilation	*n* (%)	34 (39.1)	7 (11.1)	*р* < 0.001*
Duration of mechanical ventilation, days	Me [Lq; Uq]	10.0 [6.0; 16.0]	6.0 [5.0; 8.0]	*p* = 0.046*
NICU stay, days	Me [Lq; Uq]	10.0 [6.0; 16.0]	60 [4.0; 9.0]	*р* < 0.001*
Hospital stay, days	Me [Lq; Uq]	33.0 [26.0; 41.0]	21.0 [17.0; 30.0]	*р* < 0.001*
Breastfeeding	*n* (%)	16 (18.4)	14 (22.2)	*p* = 0.353

* Statistically significant results.

Apgar scores of less than 7 points at 1st and 5th minutes were observed more often in extremely and very preterm infants (59.8% and 23.0% at the 1 and 5 min respectively, р<0.001) compared to moderately and late preterm (23.8% and 1.6% respectively, *р* < 0.001).

The clinical characteristics of preterm infants categorized by gestational age are shown in Table 1. All infants had respiratory disorders of varying severity assessed with the Silverman's score (23). Severe respiratory disorders were significantly more prevalent in extremely and very preterm infants compared to moderately and late preterm neonates (37.9% vs. 12.7%, *p* < 0.001). Forty-one children were on mechanical ventilation during the SSC intervention, with a significant predominance in the group of extremely and very preterm infants (39.1% vs. 11.1%, *p* < 0.001). The duration of mechanical ventilation was also longer in extremely and very preterm newborns compared to moderately and late preterm [10.0 (6.0; 16.0) days vs. 6.0 (5.0; 8.0) days, *p* = 0.046].

### Neonatal stress in NICU

Level of neonatal stress before and after skin-to-skin contact in preterm infants was evaluated by measuring salivary cortisol and urinary melatonin levels. The mean pre-intervention salivary cortisol level was 0.294 [0.111; 0.854] μg/dL. It was significantly higher in extremely and very preterm neonates compared to moderate and late preterm [0.344 (0.135; 1.271) μg/dL vs. 0.204 (0.099; 0.546) μg/dL, *p* = 0.028]. It was higher in children with Apgar scores below 7 at the 5th minute compared to those, who had Apgar score above 7 at the 5th minute [0.801 (0.187; 2.090) μg/dL vs. 0.255 (0.111; 0.689) μg/dL, *p* = 0.041]. Infants who were on mechanical ventilation had higher cortisol levels compared to non-ventilated newborns [0.602 (0.215; 2.090) μg/dL vs. 0.211 (0.095; 0.657) μg/dL, *p* = 0.005]. Additionally, infants who were suffering from sepsis during the study period had significantly higher pre-intervention cortisol level [1.118 (0.150; 2.250) μg/dL] compared to those who did not have neonatal sepsis [0.252 (0.111; 0.657) μg/dL, *p* = 0.005]. Baseline cortisol level did not differ in males and females [0.355 (0.114; 1.118) μg/dL and 0.211 (0.103; 0.637) μg/dL], *p* = 0.238.

The mean pre-intervention melatonin level was 4.01 [2.48; 6.34] ng/mL]. It was lower in children with Apgar scores below 7 at the 1st minute compared to those who had Apgar score above 7 at the 1st minute [3.61 (2.41; 5.46) ng/mL vs. 4.58 (2.96; 7.25) ng/mL, *p* = 0.032]. It was lower in males [3.92 (2.41; 5.68) ng/mL] compared to females [4.57 (3.09; 6.98) ng/mL], but with no statistical significance (*p* = 0.062).

**Figure 1:**
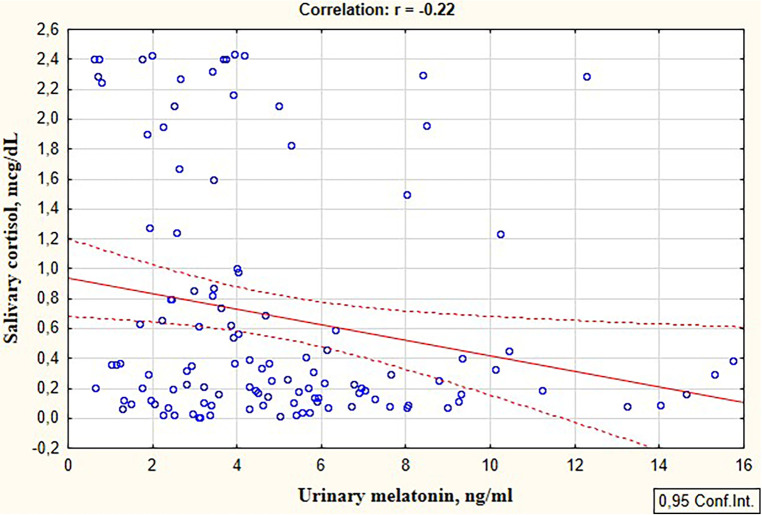
Correlation between the salivary cortisol and urinary melatonin levels in preterm infants in the study group.

An inverse correlation was revealed between the level of the stress hormone cortisol and the anti-stress hormone melatonin in preterm infants (*r* = −0.22; *p* = 0.018), Figure 1.

**Figure 2:**
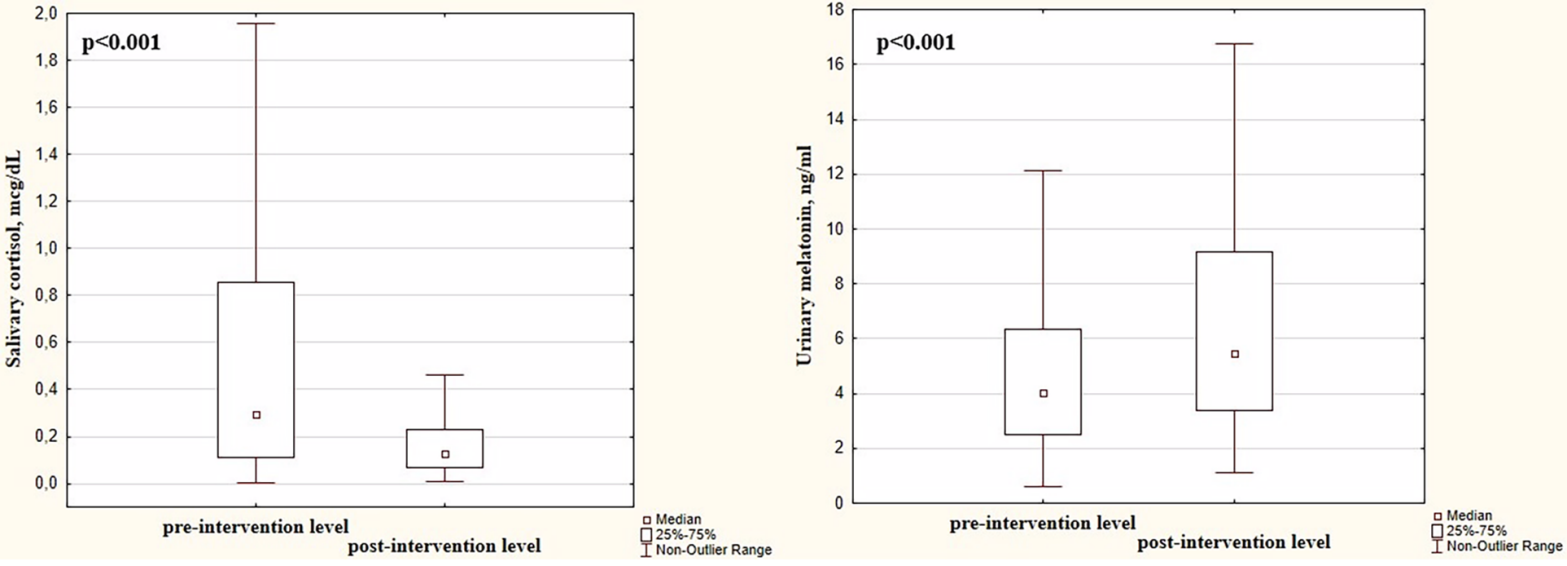
Pre- and post-intervention levels of salivary cortisol and urinary melatonin in preterm infants.

We observed a significant decrease in salivary cortisol and an increase in urinary melatonin following the SSC (cortisol pre-intervention level: 0.294 (0.111; 0.854) μg/dL vs. post-intervention level: 0.127 (0.070; 0.229) μg/dL, *p* < 0.001 and melatonin pre-intervention level: 4.01 (2.48; 6.34) ng/mL vs. post-intervention level: 5.48 (3.39; 9.17) ng/mL, *p* < 0.001]. Both cortisol and melatonin levels are shown in Figure 2.

**Figure 3:**
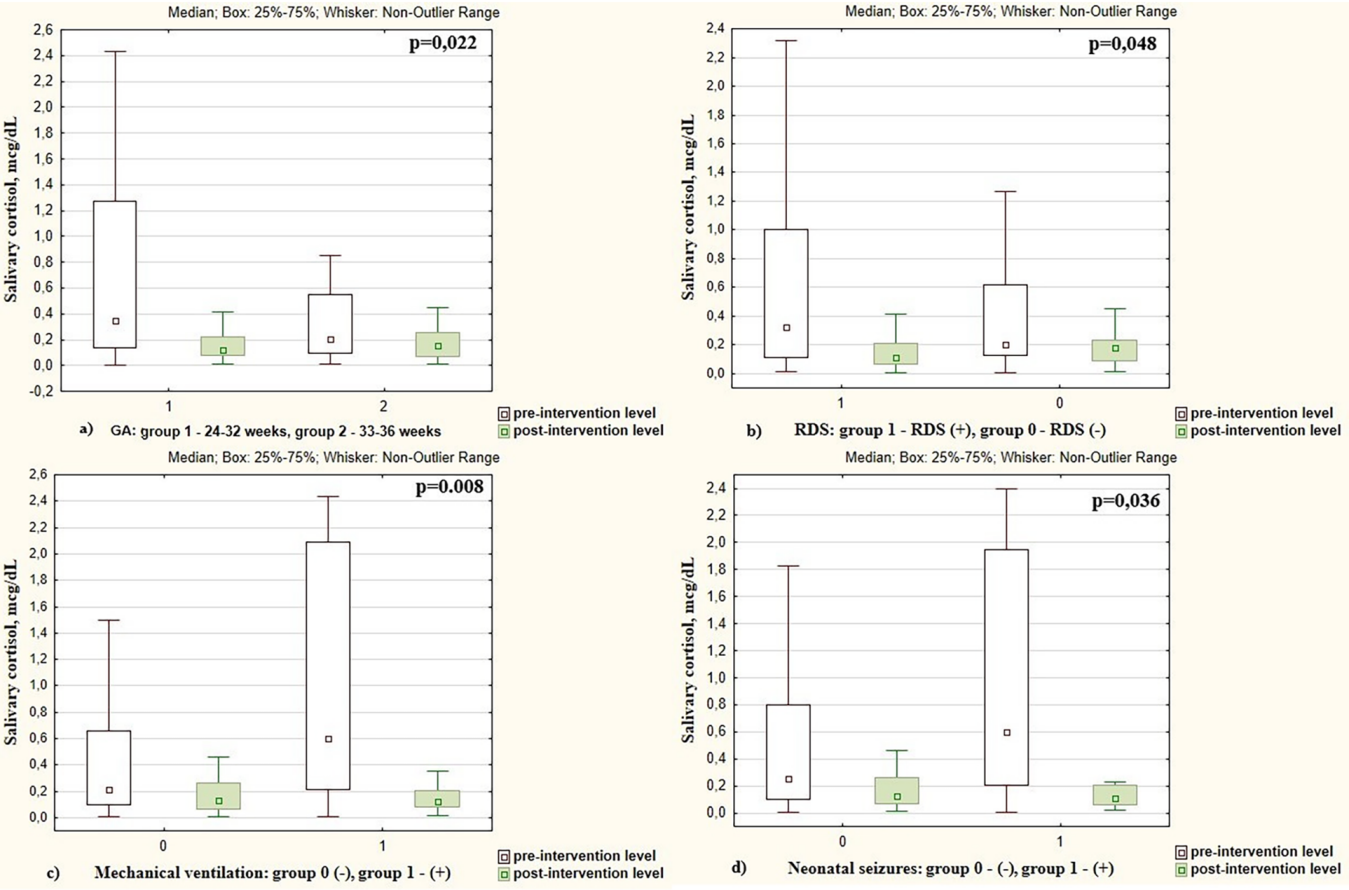
Pre- and post-intervention salivary cortisol levels in preterm infants depending on the **(a)** gestational age; **(b)** presence of respiratory distress syndrome (RDS); **(c)** mechanical ventilation; **(d)** presence of neonatal seizures.

A greater reduction in cortisol levels following skin-to-skin contacts was observed in preterm infants with a lower GA. Specifically, cortisol levels after the intervention decreased by 2.53 times in infants with GA < 32 weeks, compared to 1.56 times decrease in those with a GA more than 32 weeks (*p* = 0.022), as shown in Figure 3a. A more intensive decrease in cortisol level after skin-to-skin contact was observed in boys compared to girls (by 2.43 vs. 1.46 times, respectively, *p* = 0.012).

Changes in pre- and post-intervention cortisol levels were also associated with respiratory distress syndrome (RDS) in preterm infants. After SSC, cortisol decreased by 2.28 times in infants with RDS vs. 1.56 times in infants who did not have RDS (*p* = 0.048), Figure 3b. The cortisol level decreased more intensively in infants who were on mechanical ventilation compared to non-ventilated neonates (7.03 times vs. 1.69 times, *p* = 0.008), Figure 3c.

Cortisol level decreased more intensively in infants with neonatal seizures (confirmed clinically and electroencephalographically) during the study period. There was cortisol reduction of 7.80 times in those who experienced seizures compared to a 1.76 times decrease in infants without seizures (*p* = 0.036), as shown in Figure 3d. The pre- and post-intervention cortisol levels in preterm infants depending on demographic and neonatal factors are presented in Table 2.

**Table 2:** Pre- and post-intervention salivary cortisol and urinary melatonin levels in preterm infants depending on different demographic and neonatal factors.

Factors		Salivary cortisol, μg/dL		*p*	Urinary melatonin level, ng/ml		*p*
		Pre-intervention level	Post-intervention level		Pre-intervention level	Post-intervention level	
GA	<32 weeks	0.344 [0.135; 1.271]	0.119 [0.078; 0.219]	*р* < 0.001*	3.92 [2.45; 5.82]	5.56 [3.41; 9.43]	*р* < 0.001*
	>32 weeks	0.204 [0.099; 0.546]	0.150 [0.067; 0.258]	*p* = 0.001*	4.29 [2.79;7.25]	5.24 [3.19; 8.53]	*p* = 0.237
Gender	Male	0.355 [0.114; 1,188]	0.112 [0,064; 0.202]	*р* < 0.001*	3.92 [2.41; 5.68]	5.31 [3.10; 9.09]	*p* = 0.002*
	Female	0.211 [0.103; 0.637]	0.150 [0.078; 0.258]	*р* < 0.001*	4.57 [3.09; 6.98]	5.68 [3.39; 9.43]	*p* = 0.080
Apgar at 1st min	<7 points	0.352 [0.160; 1.599]	0.126 [0.080; 0.224]	*р* < 0.001*	3.61 [2.41; 5.46]	5.65 [3.68; 9.60]	*р* < 0.001*
	>7 points	0.264 [0.087; 0.624]	0.132 [0.067; 0.256]	*р* < 0.001*	4.58 [2.96; 7.25]	4.84 [2.98; 8.35]	*p* = 0.558
Apgar at 5th in	<7 points	0.801 [0.187; 2.090]	0.119 [0.081; 0.206]	*p* = 0.005*	2.53 [1.59; 6.05]	5.25 [3.37; 9.38]	*p* = 0.060
	>7 points	0.255 [0.111; 0.689]	0.129 [0.067; 0.236]	*р* < 0.001*	4.16 [2.79; 6.34]	5.57 [3.39; 9.09]	*p* = 0.005*
RDS	Yes	0.323 [0.111; 1.002]	0.111 [0,069; 0.212]	*р* < 0.001*	4.004 [2.44; 6.15]	5.34 [3.36; 9.09]	*p* = 0.003*
	No	0.204 [0.130; 0.615]	0.179 [0,088; 0.236]	*p* = 0.003	4.28 [3.09; 6.87]	6.46 [3.43; 11.79]	*p* = 0.101
Mechanical ventilation	Yes	0.602 [0.215; 2.090]	0.119 [0,078; 0.202]	*р* < 0.001*	3.68 [2.48; 6.84]	5.65 [3.10; 9.60]	*p* = 0.052
	No	0.211 [0.095; 0.657]	0.129 [0,068; 0.261]	*р* < 0.001*	4.16 [2.47; 6.13]	5.48 [3.45; 9.09]	*p* = 0.006*
Neonatal sepsis	Yes	1.118 [0.150; 2.250]	0.147 [0.082; 0.212]	*p* = 0.002	3.94 [1.92; 4.98]	11.30 [5.65; 14.37]	*p* = 0.002*
	No	0.252 [0.111; 0.657]	0.125 [0.068; 0.231]	*р* < 0.001*	4.16 [2.50; 6.76]	5.17 [3.34; 8.46]	*p* = 0.028*
Neonatal seizures	Yes	0.602 [0.203; 1.950]	0.112 [0.062; 0.206]	*p* = 0.003*	3.26 [2.34; 5.87]	5.42 [2.94; 8.97]	*p* = 0.170
	No	0.252 [0.103; 0.798]	0.129 [0.070; 0.258]	*р* < 0.001*	4.28 [2.57; 6.70]	5.48 [3.41; 9.17]	*p* = 0.003*

* Statistically significant results.

Changes in pre- and post-intervention melatonin levels were not associated with the GA of infants (melatonin level increased by 1.4 times after the intervention in infants with GA < 32 weeks and by 1.1 times in neonates with GA > 32 weeks), *p* > 0.05.

**Figure 4:**
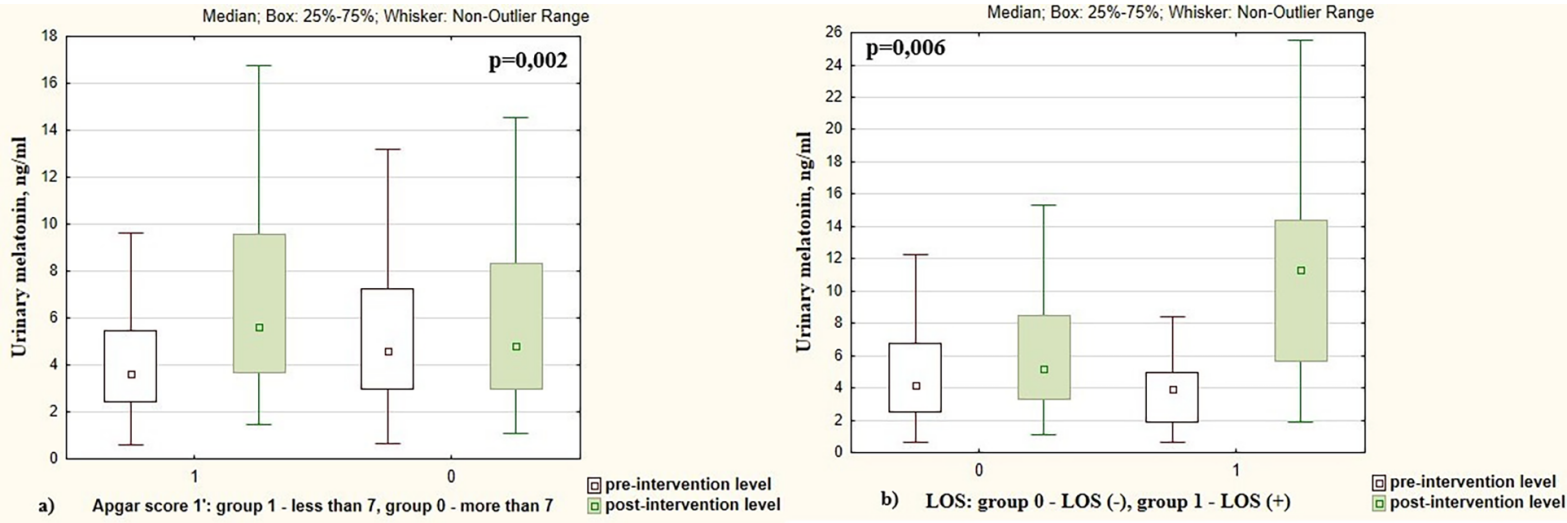
Pre- and post-intervention urinary melatonin levels in preterm infants depending on the **(a)** Apgar score; **(b)** presence of late-onset sepsis (LOS).

Melatonin levels increased more significantly in infants who had an Apgar score at 1st minute <7 points compared to newborns who had an Apgar score >7 points (2.23 times vs. 1.17 times, *p* = 0.002), Figure 4a. Changes in pre- and post-intervention melatonin levels were also associated with neonatal infection. Melatonin increased by 2.73 times in infants with late-onset sepsis (LOS) compared to 1.17 times increase in newborns without LOS (*p* = 0.006), Figure 4b. The pre- and post-intervention melatonin levels in preterm infants depending on demographic and neonatal factors are presented in Table 2.

## Discussion

The problem of neonatal stress in the intensive care unit necessitates a search for evidence-based interventions that would effectively reduce stress and its negative consequences for preterm infants. Skin-to-skin contact, promotion of a healing environment with reduction of environmental stressors, protection of infant's sleep, pain management, feeding support with breastfeeding encouragement, parents’ integration in their child's care, clustering the nursing and medical procedures are examples of ways to help reduce stress for NICU infants (24). Among its many benefits, skin-to-skin contact is recognized for minimizing the negative impact of stress from the NICU environment and from mother-child separation in the NICU (25–27).

This was the first national and one of the first international research studies to examine hormonal stress marker levels in preterm infants during NICU treatment, evaluate the impact of skin-to-skin contact on stress, and assess its effectiveness across various neonatal factors.

Our research showed a strong association between regular and prolonged SSC and reduced neonatal stress as demonstrated by decreased salivary cortisol and increased urinary melatonin levels. The obtained results were consistent with the research by other authors who studied the stress-regulatory effect of SSC (27–30). The study of Vittner et al. (28), showed that SSC reduced stress markers, including cortisol, in preterm infants, increasing overall physiological stability. Gitau et al. (29) found that cortisol levels in preterm infants decreased by 70% within 20 min of starting SSC. El-Farrah et al. (27) examined the effectiveness of SSC for 60 and 120 min per day for 7 consecutive days in preterm infants compared with usual neonatal care and found that salivary cortisol levels were significantly reduced in both SSC groups. Lyngstad et al. (30) showed that preterm infants had much lower levels of nappy changing stress if they were in SSC with their parents compared to those who were in the incubator or crib.

Uvnas-Moberg et al. (25) found that regular and repeated episodes of SSC could produce a continuous stress-buffering anxiolytic effect for preterm infants in the intensive care unit, while the deprivation of pleasant maternal touch in newborns can lead to toxic stress, which is associated with a number of developmental disorders in infants (31). That is why Professor Nils Bergman's famous saying: “Never separate a mother and her newborn baby. The benefits are even more important for preterm infants” (31), should be taken as a basis for routine neonatal practice.

To analyze the effectiveness of SSC in relation to various neonatal factors, we firstly examined the specific stress markers in preterm infants. Elevated pre-intervention cortisol levels and reduced pre-intervention melatonin levels indicated that preterm neonates in the NICU were experiencing severe stress characterized by hormonal imbalance. Cortisol is one of the several hormones produced by the adrenal glands in response to stress in infants, contributing to the adaptation to stress. Although stress hormones can offer a short-term protective effect in infants, such as maintaining blood pressure and blood sugar levels, prolonged stress exposure can lead to changes in organ function, worsen the newborn's overall condition, and slow their growth and development (32, 33). Melatonin, a neurohormone secreted by the pineal gland, possesses antioxidant and anti-inflammatory properties and is known for its calming and anti-stress efficacy. Higher levels of melatonin are associated with improved sleep quality and reduced overall stress (34).

Many authors have studied stress in preterm infants by measuring the stress hormone cortisol, and only a small number of publications have presented studies of cortisol levels in combination with stress-buffering markers, particularly oxytocin (35). Our study found an inverse correlation between the stress hormone cortisol and the anti-stress hormone melatonin in preterm infants in the NICU (*r* = −0.22; *p* = 0.018). This confirmed the relationship between chronic stress and melatonin secretion. It is also known that chronic stress to which newborns are exposed in intensive care units leads to activation of the hypothalamic-pituitary-adrenal system, which affects melatonin secretion (36).

A detailed analysis of stress markers revealed that cortisol level was associated with the infants’ gestational age. Cortisol was significantly higher in extremely and very preterm neonates compared to moderate and late preterm newborns. This finding indicated an increased stress response in the NICU among the most premature children, which was aligned with the results reported by other authors (37, 38). In particular, van Dokkum et al. (37) showed a similar association of chronic stress with GA in preterm infants assessed using the Neonatal Infant Stressor Scale. Additionally, Urfer et al. (38) found that extremely preterm infants (born before 28 weeks) had significantly higher cortisol levels compared to very preterm neonates (born at 28–32 weeks), as well as moderately and late preterm infants (32–37 weeks). This indicates a significant stress response gradient relative to the degree of prematurity (39).

We found that cortisol levels were also higher in children with Apgar scores below 7 points, while melatonin levels were significantly lower in infants with low Apgar scores. These results indicated high levels of stress in infants with low Apgar scores and were consistent with the reports of others researchers (37). Razaz et al. (40) showed that melatonin levels were lower in newborns with an Apgar score below 7 points at the first minute. Preterm infants with low Apgar scores receive primary resuscitation in the delivery room and intensive neonatal care during the first hours of life (32, 41), which can serve as an additional trigger for an excessive long-lasting stress response. It was shown that the rate of mortality, morbidity, and therefore the frequency of neonatal interventions and complications are significantly higher in preterm newborns with low Apgar scores (39, 42). The association of low Apgar scores with lower melatonin levels may also be explained with reduced neuroendocrine activity of the pineal gland in conditions of impaired adaptation against the background of preterm infants’ immaturity (43, 44).

It was revealed that mechanical ventilation of premature babies was reliably associated with an increased level of cortisol. The obtained results were consistent with those of other authors who noted that invasive ventilation is a stressful experience for newborns and leads to changes in neuroendocrine parameters, pain indicators and physiological reactions (45). This stress response is exacerbated by the invasive nature of the procedure, which involves endotracheal intubation causing both physical and psychological stress for the newborn (46). Although, there are no data on how painful and stressful invasive lung ventilation itself is, it is accompanied by a large number of potentially painful interventions, such as intubation, reintubation, frequent endotracheal aspirations, and skin damage due to changes in adhesive materials (47).

Our study revealed an association between pre-intervention cortisol level with neonatal sepsis. These findings were consistent with Soliman et al. (48), who found a significantly higher level of cortisol in newborns with sepsis (3-fold increase) and RDS (2.3-fold increase) compared to healthy neonates, explaining this as an adaptive, compensatory response. However, high and prolonged levels of cortisol during acute and chronic stress contribute to the dysregulation of allostatic processes that mediate inadequate adaptation to stress (32).

We found a greater reduction in cortisol levels after skin-to-skin contacts in preterm infants with a lower gestational age, in males compared to females, and in those who had RDS, neonatal seizures, and required ventilator support. At the same time, the melatonin level increased more significantly in infants who had low Apgar score and neonatal sepsis. These neonatal factors were associated with hormonal imbalances in preterm infants, accompanied by high pre-intervention cortisol levels and low pre-intervention melatonin levels. Therefore, regular and long-lasting SSC is especially important for the most vulnerable preterm infants in the NICU.

Bergman and Bergman (49) showed that the mother's body is the most optimal environment that ensures the physiological stability of the infant and contributes to the maturation of his (her) own regulatory processes. Improvement of physiological stability in infants who have SSC is associated with reduced stress in response to this intervention (31). It is known that RDS, sepsis, neonatal seizures, and low Apgar scores are often accompanied by instability of physiological parameters, which is why SSC with its stress-buffering properties is so effective in newborns who have these adverse neonatal factors. Lee et al. (50) showed that SSC reduced work of breathing compared to incubator care in mechanically ventilated preterm infants. They indicated that SSC among ventilated preterm infants was not only safe but also stabilized and improved their respiratory physiology. These beneficial effects related to SSC were more evident during invasive than non-invasive ventilation. Kato et al. (51) also found that respiratory efforts were significantly reduced during SSC in ventilated preterm infants.

Historically, SSC has primarily been implemented for preterm infants. The importance of SSC for preterm infants with a lower gestational age was confirmed in the randomized control trial conducted by Kristoffersen et al. (52). The authors revealed that SSC for very preterm infants following delivery is safe and feasible both for vaginal and caesarean deliveries. These studies highlight the importance of implementation of skin-to-skin contact for extremely and very preterm neonates.

## Practical implementation

The WHO recommends a non-separation strategy between the mother and infant from birth regardless of GA (53), and our study supports this change in clinical practice for very fragile preterm infants. Preterm neonates, especially those who are extremely and very preterm, with respiratory disorders, neonatal sepsis, neonatal seizures, low Apgar scores and those with mechanical ventilation should not be denied the benefits from skin to skin contact starting in the NICU. This intervention is even more effective for these infants than for healthy preterm neonates. Considering the potential benefits of this relatively simple intervention, clinicians should implement SSC as a stress reducing strategy.

## Conclusion

The reduction in cortisol levels and the increase in melatonin levels provided strong evidence that skin-to-skin contact ameliorated the NICU-related stress in preterm infants. A greater reduction in cortisol levels in response to regular and long-lasting skin-to-skin contacts was associated with the gestational age, gender, RDS, neonatal seizures, mechanical ventilation. Meanwhile the increase in melatonin levels was associated Apgar score and neonatal sepsis.

We found higher indicators of stress and more dramatic responses to SSC in reducing indicators of stress in infants with lower GA than in infants with higher GA, indicating that SSC may be even more important for lower GA infants. The infants who need SSC the most should not be denied the care they need to reduce the stress they experience from being born too soon and continuing their gestational development in the stressful environment of the NICU.

## Data Availability

The raw data supporting the conclusions of this article will be made available by the authors, without undue reservation.
